# Constitutional chromothripsis involving the critical region of 9q21.13 microdeletion syndrome

**DOI:** 10.1186/s13039-015-0199-3

**Published:** 2015-12-18

**Authors:** Rita Genesio, Paolo Fontana, Angela Mormile, Alberto Casertano, Mariateresa Falco, Anna Conti, Adriana Franzese, Enza Mozzillo, Lucio Nitsch, Daniela Melis

**Affiliations:** Department of Molecular Medicine and Medical Biotechnology, Federico II University, Naples, Italy; Department of Translational Medical Sciences, Section of Pediatrics, Federico II University, Naples, Italy

**Keywords:** Chromothripsis, 9q21.13 deletion syndrome, *GNAQ*

## Abstract

**Background:**

The chromothripsis is a biological phenomenon, first observed in tumors and then rapidly described in congenital disorders. The principle of the chromothripsis process is the occurrence of a local shattering to pieces and rebuilding of chromosomes in a random order. Congenital chromothripsis rearrangements often involve reciprocal rearrangements on multiple chromosomes and have been described as cause of contiguous gene syndromes. We hypothesize that chromothripsis could be responsible for known 9q21.13 microdeletion syndrome, causing a composite phenotype with additional features.

**Case presentation:**

The case reported is a 16- years-old female with a complex genomic rearrangement of chromosome 9 including the critical region of 9q21.13 microdeletion syndrome. The patient presents with platelet disorder and thyroid dysfunction in addition to the classical neurobehavioral phenotype of the syndrome.

**Conclusions:**

The presence of multiple rearrangements on the same chromosome 9 and the rebuilding of chromosome in a random order suggested that the rearrangement could origin from an event of chromthripsis. To our knowledge this is the first report of congenital chromothripsis involving chromosome 9. Furthermore this is the only case of 9q21.13 microdeletion syndrome due to chromothripsis.

## Background

The chromothripsis is a newly recognized biological phenomenon, first observed in tumours [1] and then rapidly described in congenital disorders [2, 3]. The principle of the chromothripsis process is the occurrence of a local shattering to pieces of chromosomes in an unique catastrophic cellular event, followed by a reassembly of the pieces into one or more chaotic derivative chromosomes by DNA repair processes, most likely non-homologous end joining [4]. Some pieces may be lost or combined in small circular extra-chromosomes (double-minute chromosomes).

The chromothripsis rearrangements found in cancer are characterised by high copy number changes [1]; in contrast, a striking characteristic of congenital chromothripsis rearrangements is their relatively balanced state, despite the presence of multiple DNA breaks across several chromosomes [5, 6]. This does not mean that unbalanced chromothripsis does not occur in germline or during post-zygotic divisions, but it strongly emphasizes the effect of selection as a bias factor in the assessment of congenital chromothripsis, since only balanced chromothripsis outcomes compatible with life have been found in individuals to date.

Next-generation sequencing and FISH experiments have been performed to characterise congenital chromothripsis-associated complex chromosomal rearrangements involving chromosomes 1, 2, 3, 5, 7, 8, 13, 15,19, X [5–8]. Chromothripsis events have been described as cause of contiguous gene syndromes such as Cri-du-chat syndrome [9] and 16q21-q22.1 deletion [3] and in disruption of the FOXP2 (forkhead box P2) gene [7].

To our knowledge, congenital chromothripsis involving chromosome 9 has not yet been reported, as well as chromothripsis events have never been associated to the 9q21.13 microdeletion syndrome.

Here, we report a case of a girl with severe intellectual disability, epilepsy, platelet dysfunction, dysmorphisms and hypothyroidism, carrying a complex chromosomal rearrangement, which involves the critical region of the 9q21.13 microdeletion syndrome. Although many phenotypic signs may be ascribed to this syndrome, the proband presents also with other features, suggesting that the specific phenotype might be the result of the very complex rearrangement caused by chromothripsis. We here describe the molecular characterisation of the rearrangement, analysing possible role of the genes included in it and speculating about a genotype–phenotype correlation.

## Case presentation

### Clinical report

**Fig. 1:**
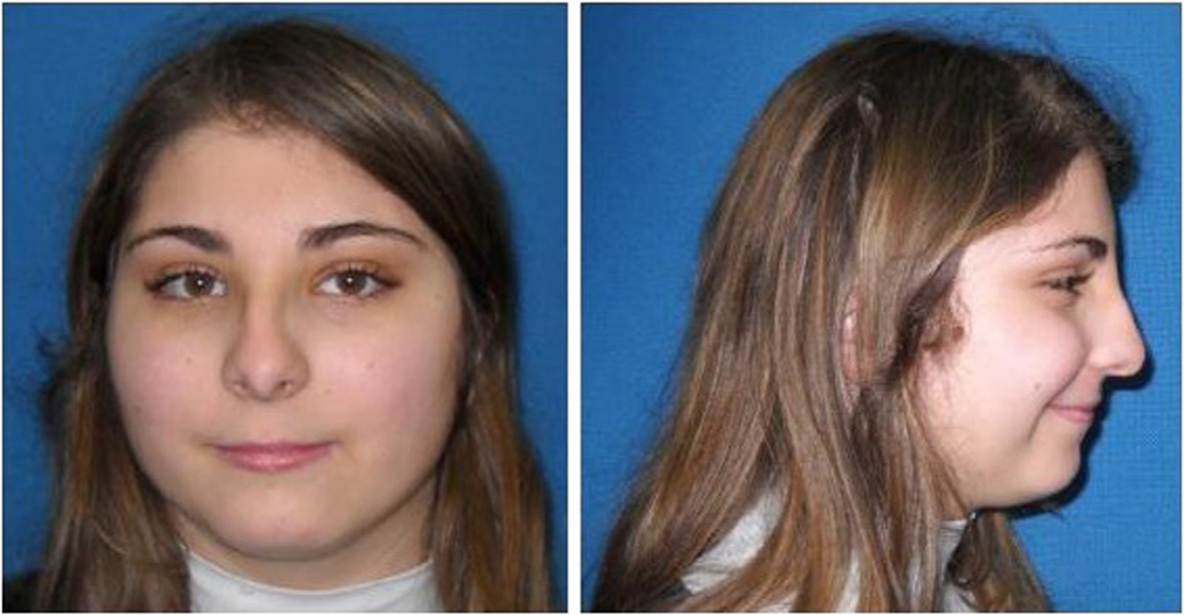
Facial phenotype of the patient at the age of 16

We report the case of a 16-years-old female patient. She was born at term by vaginal delivery. At birth she weighted 2.780 Kg (10° - 25° ct), her length was 48 cm (25°-50°) and her head circumference 31.7 cm (<3°ct); she had an APGAR score of 8 at 1’ and 9 at 5’. At the age of 6 months she showed developmental delay, moderate hypertonia of limbs and motor development impairment. When she was 7 years old, an impaired platelet aggregation was detected. At the age of 10 years she had seizures with revulsion of the eyeballs, hypertonia, enuresis, post-critical sleep and vomit and received a diagnosis of “secondarily generalized partial epilepsy”. We examined the proband at the age of 16 years, when dysmorphic features were visibly evident (Fig. 1), including downslanted palpebral fissures, long eyelashes, high palate, pronounced columella, retrognatia and clinodattily of the 5th finger, irregular shaped CLS on the left flank; the patient also had malformed, hypoplastic inner labia, visible only on the superior tract. X-ray examination demonstrated a flattening of the physiological cervical and lumbar lordosis and of the dorsal kyphosis. A diagnosis of subclinic hypothyroidism was performed on the basis of thyroid hormone profile (FT3: 2,8 pg/ml; FT4: 0,9 ng/dl; TSH: 9,2 μU/ml) and thyroid ultrasound showed the presence of an iso-hypo echogenic nodule with maximal diameters of 10 and 9 mm, with no neoplastic nature, as shown at the cytological examination. The last I.Q. evaluation, performed at the age of 16 by the WAIS-R scale, attested a moderate cognitive impairment (total I.Q 46, Verbal I.Q < 46, Performance I.Q 46).

### Methods

Cytogenetic analysis was performed on GTG-banded metaphases from lymphocytes at a resolution of 500 bands approximately according to the standard cytogenetic protocol. The description of karyotypes was made in accordance with the ISCN 2013 (International System of Human Cytogenetic Nomenclature).

FISH analysis using Satellite Enumeration Probes (Kreatech Diagnostics, Amsterdam, NL) and bacterial artificial chromosome probes (BAC) were done according to standard procedures. The BAC probes were selected from the UCSC genome browser [10] and provided by Professor M. Rocchi (rocchi@biologia.uniba.it). Multicolour banding (MCB) was performed using the multicolour banding DNA Probe Kit based on microdissection derived region-specific libraries for chromosome 9 (Meta-Systems, Arese MI). Fluorescent images were analysed using a fluorescence microscope (AxioImager.Z1 mot, Zeiss) with ISIS software imaging system (MetaSystems, Altlussheim, Germany) for image capturing and processing. A NimbleGen CGX-6 PKI Array chip with a resolution of 175 kb in the backbone and 50 kb in the targeted region (Roche NimbleGen, Inc.,Madison,WI, USA) was used in accordance with manufacturer’s guidelines for the whole genome CGH analysis. The arrays were analyzed using Genoglyphix® software 3.0 (PerkinElmer Spokane, WA), referring to Hg19 Genome Assembly (GRCh37/hg19). Copy number variations were classified according to the Database of Genomic Variants [11], the DECIPHER Database [12] and the UCSC Genome Browser [10].

### Results

**Fig. 2:**
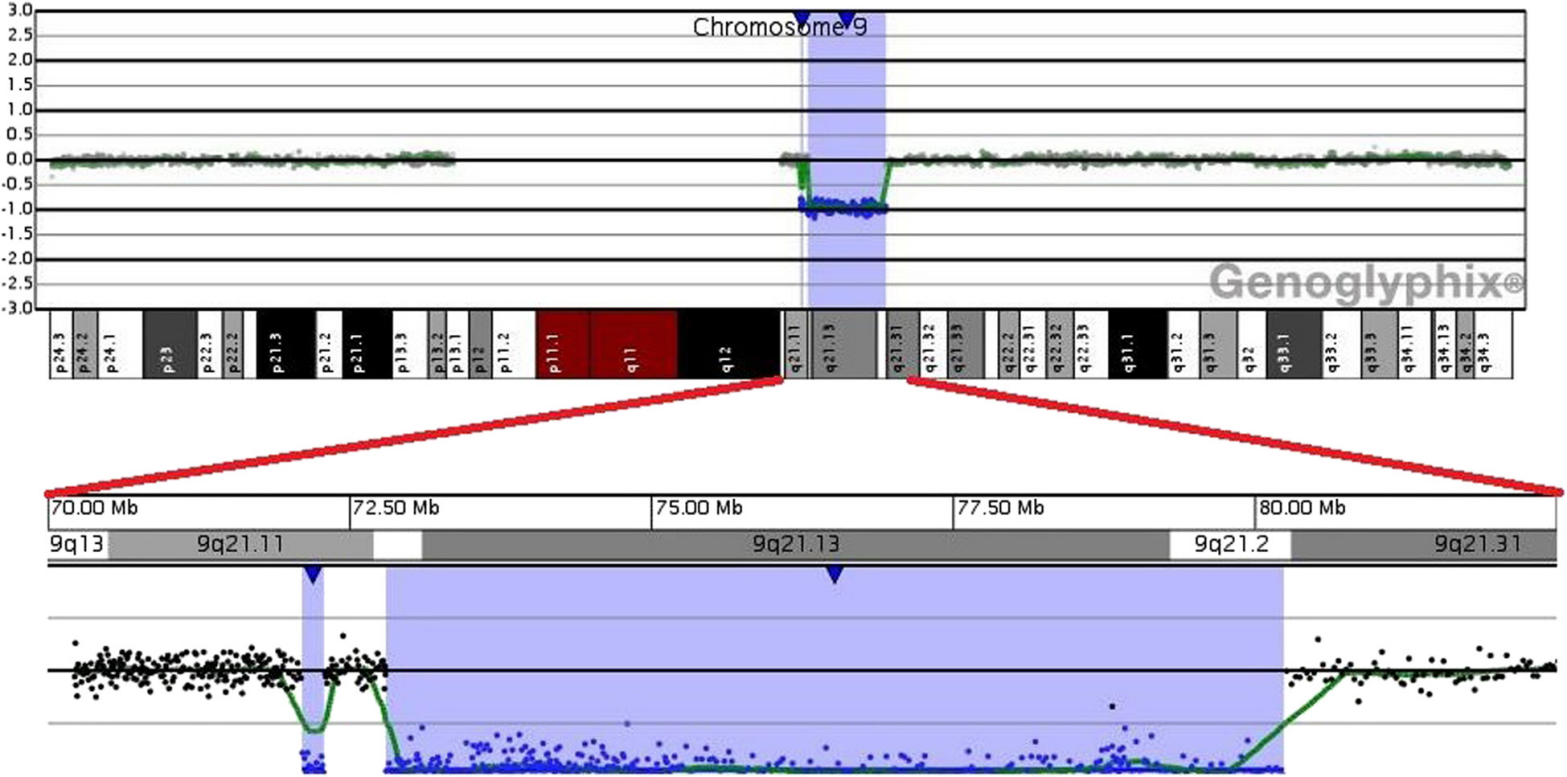
Array-CGH analysis showing a 9q21.11 deletion, spanning approximately 176.56 kb and a 9q21.12q21.2 deletion, spanning approximately 7.44 Mb

**Fig. 3:**
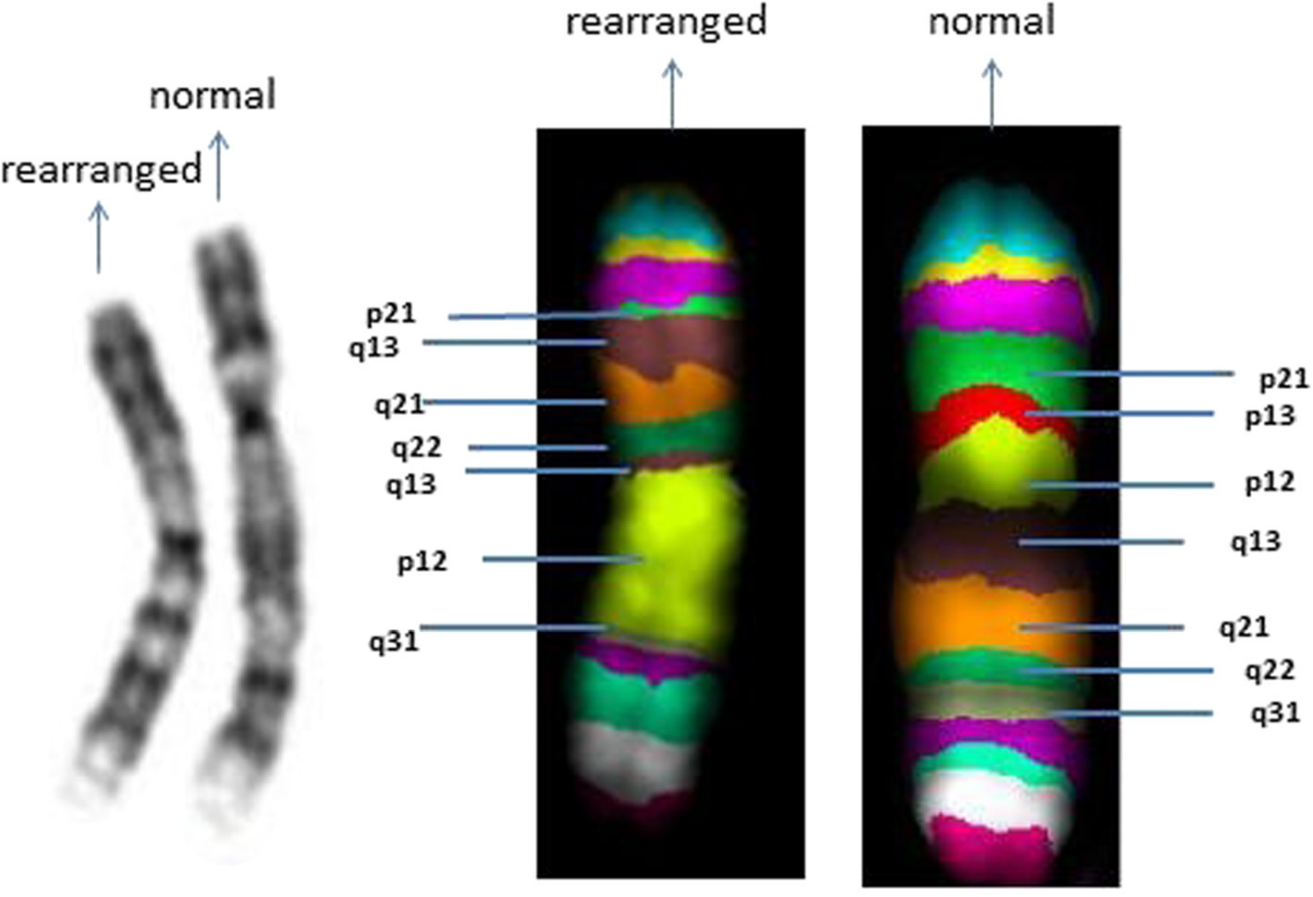
G-Banding image of rearranged chromosomes 9 (*on the left*) and of the normal chromosome 9 (*on the right*) and Multicolour banding image of the rearranged chromosome 9 (*on the left*) compared to the normal chromosome 9 (*on the right*) shows bands involved in the rearrangement

**Fig. 4:**
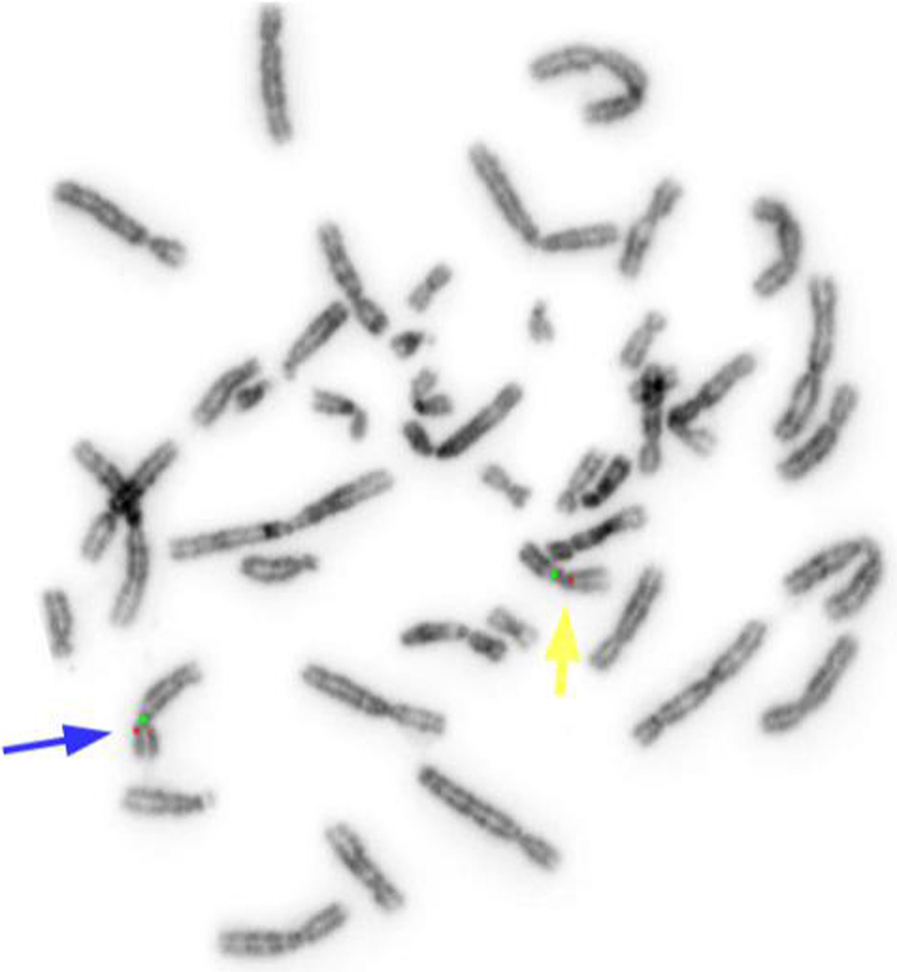
Dual-colour-FISH with centromeric probe (*green*) for chromosomes 9 and BAC probe RP11-182N22 (*red*) mapping in 9p13.3 region showed that the BAC probe signal was present in the q arm of the der9 (*yellow arrow*), instead of the p arm of chromosome 9 (*blue arrow*)

Cytogenetic analysis revealed an abnormal female karyotype with 46 chromosomes and a chromosomal rearrangement involving one chromosome 9 (der(9)) in all the examined cells. Parental karyotypes were normal. The array analysis identified a 9q21.11 deletion, spanning approximately 176.56 kb and a 9q21.12q21.2 deletion, spanning approximately 7.44 Mb (Fig. 2). The derivative chromosome 9 characterised by Multicolour banding (MCB) showed an anomalous assembly of the bands from the 9p21 band to 9q31 band (Fig. 3), even though MCB 9 was not able to identify the 9p13 region on der(9) perhaps because of a fluorescence overlapping. Therefore the der(9) was further characterised by dual-colour-FISH using both the Satellite Enumeration probe for chromosomes 9 (SE 9) and the BAC probe RP11-182N22 mapping to 9p13.3 region. This FISH showed an inversion not revealed by MCB (Fig. 4), as the BAC probe signal was present in the q arm, instead of the p arm, of one chromosome 9.

The complete chromosomal characterisation according to ISCN 2013 was as follows:

46,XX,inv(9)(p13q21.1).ish inv(9)(9pter- > p21::q13- > q22::q13- > p13::q31- > qter)(wpc9+, pcp9+,RP11-182N22+, D9Z1+).arr[hg19]9q21.11(72110094–72286657)x1,9q21.12q21.2(72803705–80243747)x1.

## Discussion

The term chromothripsis was introduced to indicate catastrophic DNA rearrangements in cancer genomes [1]. More recently, a very similar chromosome shattering process has been described in constitutional rearrangements [5–7].

Congenital chromothripsis rearrangements often involve reciprocal rearrangements on multiple chromosomes. Furthermore the number of breakpoints for chromothripsis rearrangements in the germline is substantially lower than t that observed in cancer [5].

Recently, whole-genome sequencing and FISH analysis led to the discovery of two other classes of complex catastrophic chromosomal rearrangements; namely chromoanasynthesis and chromoplexy [13, 14]. Although the underlying mechanisms for these phenomena are unknown and likely different, all of these patterns of rearrangements appear to have originated from a single catastrophic event. Chromoanasynthesis, as well as chromothripsis, affects limited portions of the genome (often a single chromosome or chromosome arm) and is characterised by a copy number profile alternating loss and gain of DNA tracts. In contrast to chromothripsis, chromoanasynthesis reflects resynthesis of segments from one chromatid. Chromoanasynthesis also contains frequent insertions of short sequences between the rearrangement junctions that are copied from the rearranged segments (templated insertions). Chromoplexy is characterised by a closed chain of translocations, with little or no copy number alteration.

The patient that we have described shows two deletions and a random and anomalous assembly of the chromosome bands on a single chromosome 9 (Fig. 3). Furthermore the two deleted traits have clustered breakpoints within 9q21 region interrupted by a small region without DNA gain or loss. These features led us to hypothesize that this rearrangement could be caused by congenital chromothripsis.

It is interesting to note that all the cases of congenital chromothripsis described so far present a composite phenotype, including known syndromes, due to the complex genomic rearrangements resulting from the catastrophic events [3, 7, 9].

The patient here described presents with severe intellectual disability, epilepsy, dysregulation of platelet aggregation, dysmorphisms, genitalia malformations and hypothyroidism. The array-CGH analysis demonstrated a 756 kb deletion at 9q21.11 together with a 7.4 Mb deletion at 9q21.12-q21.2, which encompasses 20 OMIM genes and includes the candidate critical region for the 9q21.13 microdeletion syndrome.

The 9q21.13 microdeletion syndrome is a recently discovered condition, characterised by intellectual disability, seizures and several aspecific dysmorphic features. A 750 kb putative critical region has been proposed [15], encompassing four genes, *RORB*, *TRPM6*, *NMRK1* and *OSTF1. RORB* (RAR-related orphan receptor B) gene is a strong candidate for neurological disorders [15], therefore its haploinsufficiency could be responsible for the severe neurological impairment observed in our patient.

Even though phenotypic signs of the proband, such as intellectual disability and epilepsy, may be mostly ascribed to 9q21.13 microdeletion syndrome, other signs, such as platelet disorder and hypotiroidism have not been described in 9q21.13 syndrome, demonstrating that a wider size of the deletion, if compared to the critical region for the 9q21.13 deletion syndrome, the different positioning of the genes included in the region, due to chromotripsis events could influence the severity of phenotypic traits of patient [16, 17].

Both platelet disorder and hypotiroidism might be to ascribed to another deleted gene, namely *GNAQ*, which maps to 9q21.2, outside the critical region of the 9q21.13 deletion syndrome. *GNAQ* encodes a guanine nucleotide binding protein, which is an alpha subunit in the Gq class. This protein is coupled with 7-transmembrane domain receptors with a GTPase intrinsic activity that mediates the induction of phospholipase C-beta into the cells [18]. In platelets, this signaling is responsible for the calcium mobilization from the endoplasmic reticulum, that mediates several pathways ending with platelet aggregation, through the phases of shape change, improvement of the secretion of activators from the granules and conversion of GP IIb/IIIa to its activated form [19]. In the first description of a human platelet G protein defect [20], similarly to our case, the patient had diminished platelet aggregation and secretion in response to multiple agonists, despite the presence of normal dense granule stores.

The same gene could be involved in the thyroid dysfunction. Mice lacking the alpha subunits of Gq and G11 specifically in thyroid epithelial cells showed a severe reduction of the iodine organification and the secretion of thyroid hormone in response to TSH, and the majority of them developed hypothyroidism within months after birth [21].

## Conclusions

In conclusion, the patient that we have described, with four rearrangements on a chromosome 9 is the first case, described so far, in which congenital chromothripsis affects just one chromosome, as well as in cancer.

This is also the first report of congenital chromothripsis involving chromosome 9 and, to our knowledge, this is the only case of 9q21.13 microdeletion syndrome due to chromothripsis.

We speculate that chromothripsis could be the basis of known contiguous gene syndromes, with additional features, causing composite phenotypes. We suggest that the patient’s specific phenotype might be the result of the very complex rearrangement, caused by chromothripsis. The different size of the deletion, if compared with the critical region for the 9q21.13 deletion syndrome, the type and the positioning of the genes involved in the rearrangement might influence the severity of the proband’s phenotype. Going forward, we will better define the features and origins of this complex chromosome rearrangement by Whole Genome Sequencing.

## Consent

Written informed consent was obtained from the patient for publication of this Case report and any accompanying images. A copy of the written consent is available for review by the Editor-in-Chief of this journal.
